# Predicting the presence of tephra layers in lacustrine deposits using spectral gamma ray data: An example from Lake Chalco, Mexico City

**DOI:** 10.1371/journal.pone.0315331

**Published:** 2024-12-30

**Authors:** Mehrdad Sardar Abadi, Christian Zeeden, Arne Ulfers, Alex Susan Meyer, Thomas Wonik, Mexidrill team

**Affiliations:** LIAG-Institute for Applied Geophysics, Hannover, Germany; Ural Federal University named after the first President of Russia B N Yeltsin Institute of Physics and Technology: Ural’skij federal’nyj universitet imeni pervogo Prezidenta Rossii B N El’cina Fiziko-tehnologiceskij institut, RUSSIAN FEDERATION

## Abstract

Spectral gamma ray borehole logging data can yield insights into the physical properties of lake sediments, serving as a valuable proxy for assessing climate and environmental changes. The presence of tephra layers resulting from volcanic ash deposition is not related to climate and environmental conditions. As a result, these layers pose challenges when attempting to analyze paleoclimate and environmental time series. Gamma rays are composed of photons, which are elementary particles of electromagnetic radiation. Tephra layers emit photons at specific energy levels that create a distinct pattern in their gamma-ray energy spectrum. The gamma-ray signature of tephra layers varies depending on the stage of the volcanic eruption. Additionally, there is a significant difference between the gamma-ray signature emitted by tephra layers and that of the background lake sediments. A composite signature can be used to predict tephra layers from background sediments by combining several gamma-ray signatures of tephra layers at different depths. We propose five-step protocol for detecting tephra layers within sediments through the utilization of gamma-ray spectroscopy. This protocol is based on a combination of physical aspects of gamma-ray spectroscopy and geological information specific to the lake system being studied. A subset of the training dataset is used, consisting of known tephra and non-tephra layers. The protocol involves identifying similarities between known tephra layers, analyzing differences in gamma-ray signals between tephra and non-tephra layers, and studying the composition of energy channels at various depths within the training dataset. Multiple linear regression models are used to predict the relationship between the composition of tephra layers as a dependent variable and the constituent energy channels of the gamma-ray signal as independent variables. The proposed protocol has the potential to accurately detect and identify thick tephra layers (> 10 cm in thickness) based on the rate of spectral gamma ray measurement in sedimentary sequences. This approach could enhance stratigraphic resolution by enabling finer subdivision of layers in an interior basin.

## Introduction

**Fig 1:**
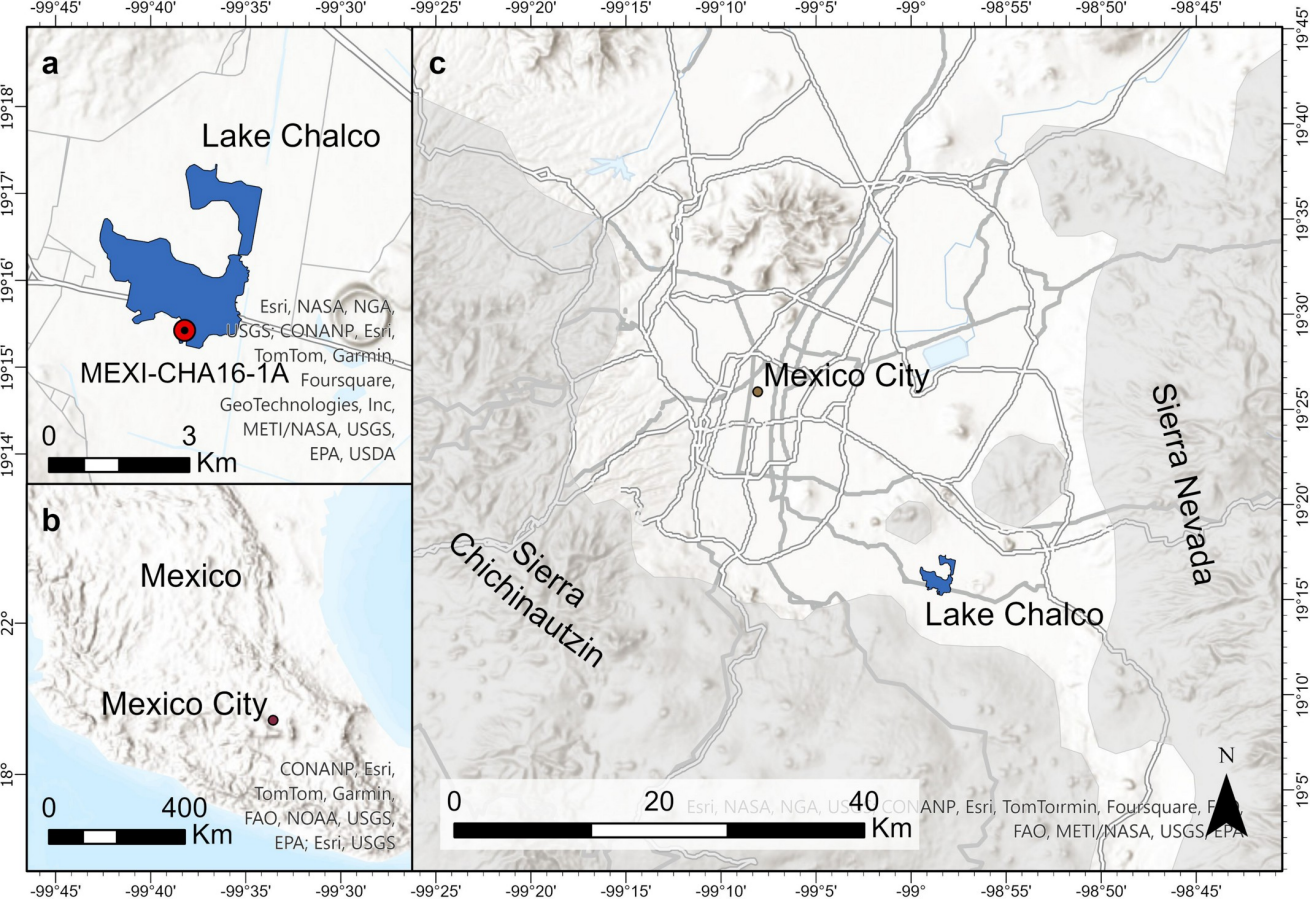
Map of Central Mexico. a) Location of the studied site (MEXI-CHA16-1A) at Lake Chalco. b) Location of Mexico City in Central Mexico. c) Location of Lake Chalco relative to the Sierra Nevada and Sierra Chichinautzin mountain ranges in Central Mexico.

The detection of gamma (γ)-radiation serves as a crucial method in identifying elemental isotopes within the geological record enabling stratigraphic correlation and facilitating paleoclimate investigations. The γ-radiation is a relevant physical property, can be measured non-destructively and affordably on core samples, directly in boreholes and on surfaces [1]. Tephra layers constitute important stratigraphic marker horizons that can be used as combined high resolution geochronological and chemical markers to constrain key stratigraphic events [2–4]. Lake sediments may contain tephra layers [5, 6], which usually influence the γ-ray signal. This is, on one hand, a great opportunity for detecting tephra, which can be used for age determination. On the other hand, these pose a challenge when assessing the non-volcanic primary signals caused by environmental and climatic agents [7]. This is especially true for lakes which formed adjacent to volcanic fields with volcanically supplied lake sediment budgets. Lake Chalco, located in Central Mexico, is an example of a lake surrounded by volcanoes, and its lacustrine sediments include volcanic ash deposits or tephra [8] (Fig 1). The γ-ray signal in sediments contains components of both background sedimentation and tephra layers.

**Fig 2:**
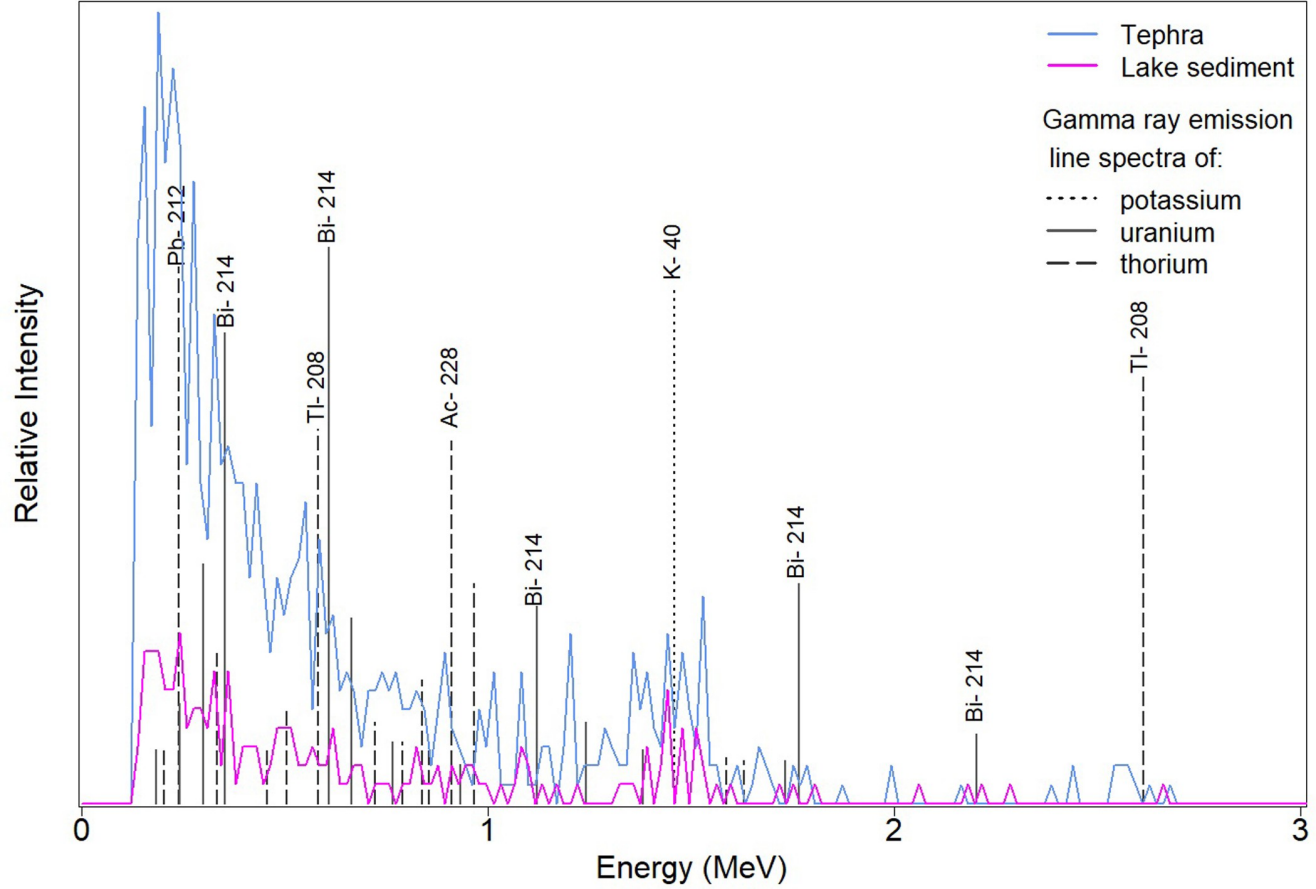
γ-ray emission line spectra of potassium, thorium and uranium. The relative intensities of isotopic peaks of potassium (^40^K) and the equilibrium decay series of uranium (^238^U and ^235^U and their daughters) and thorium (^232^Th and its daughters) are plotted. γ-ray emission of a tephra layer (blue line) at the depth of 95 m and a non-tephra layer (pink line) as a lake sediment at the depth of 88 m are illustrated for comparison.

Tephra is comprised of unconsolidated fragments ejected into the air by volcanic eruptions. Importantly they contain juvenile pyroclastic particles (e.g. ash and crystals) that consist of differing quantities of potassium-rich minerals (K), and minerals that contain elements from the uranium (U) and thorium (Th) series. The concentration of K, U and Th series in lacustrine sediments vary as a function of silicate-weathering rate in the source and loss of elements in stream water that eventually reduces the concentration of these elements compared to tephra layers [9, 10]. Natural sources of γ-radiation are potassium (^40^K), the equilibrium decay series of uranium (^238^U and ^235^U and their daughters), and thorium (^232^Th and its daughters), and thus combined γ-ray signals reflect the presence of these elements and isotopes (Fig 2). The presence of tephra layers, resulting from the deposition of volcanic ash within lake sediments, is independent of climate and poses challenges in the interpretation of paleoclimate time series.

Detecting tephra layers often necessitates costly, time-consuming, and potentially destructive tests [11, 12]. Many different techniques are used to detect tephra layers, including cryptotephras, which may not always be visible to the naked eye, in sediment cores [13–15]. The recurrent loss of sediments during drilling campaigns, which is common in unconsolidated material, increases uncertainty in identifying volcanic deposits. Core-log integration allows scientists to determine the approximate effect of tephra layers, particularly in γ-ray logs. However, core loss or the presence of many tephra layers throughout the stratigraphic record poses a challenge in editing the γ-ray log [9].

γ-ray logging depth is widely acknowledged as a more reliable representation of the entire length of lacustrine sediments compared to core depth [7, 9]. The spectral γ-ray tool is one of the few instruments capable of operating in a cased hole, thereby offering a continuous signal of γ-ray and provides insight into the distribution of K, Th and U. γ-ray spectrometry is an analytical technique that displays the energy distribution of emitted photons from source isotopes [16]. Each γ-ray photon possesses a distinct energy level representative of specific source isotopes. A γ-ray spectrometer converts detected energy into a multichannel spectral pattern ranging from 0 to 3 megaelectronvolt (MeV). Each channel represents a specific energy band of γ-ray energy in the range of 11.7 kiloelectronvolt (keV) with increasing energy levels from channel 0 to 256. The number of counted photons is recorded as the relative intensity of γ-ray emission in each channel. Potassium has a characteristic line spectra coinciding with the 1.46 MeV γ-ray emitted. However, Th and U represent a series of γ-ray emissions of decay products with distinctive energy levels (Fig 2). The total count of the energy distribution of detected photons in the spectrum gives a measure of total γ-radiation.

We hypothesize that tephra layers exhibit distinct γ-ray signals that correspond to the composition of tephra layers. The detection of embedded tephra layers can be accomplished by analyzing both the intensity of the γ-ray signal and the composition of its constituent energy channels. We examine whether the tephra layers produced by different eruptions possess different signatures through their γ-ray spectrum. In this study, we analyze the sediments of Lake Chalco in Mexico City, which were obtained through a drilling project as part of the MexiDrill project under the International Continental Scientific Drilling Program (ICDP) [8, 17]. We propose a protocol that uses γ-ray spectroscopy, and representative horizons of tephra and non-tephra from a core subsection within the same lake system to identify tephra layers embedded in other sediments. The proposed protocol reduces the effect of γ-radiation emitted from tephra layers on the preliminary climate signals recorded through the lacustrine deposits of Lake Chalco.

### Geological context

Lake Chalco is located in the vicinity of Mexico City. The formation of the lake is linked to the tectonic history of Central Mexico. The subduction of the Cocos Plate (a part of the Pacific Plate) beneath the North America Plate gave rise to a series of volcanoes in south-central Mexico approximately 16 million years ago [18]. The extension of a continuous chain of volcanic arcs on the continental crust resulted in the formation of a vast interior drainage basin nearly one million years ago. The current designation for this geological formation is known as the Basin of Mexico (Fig 1). Following the formation of the Basin of Mexico, the retention of water within this basin resulted in the formation of several lake systems, encompassing approximately 1500 km^2^ of the basin floor at an elevation of 2240 m above sea level [8]. The drainage of the basin was the main source of present-day lake sediments, which moved from encircled volcanic crests through the river systems. The volcanic rock contains primary silicate minerals with mainly plagioclase and a variety of ferromagnesian minerals, which are the primary source of the radiogenic elements [9, 19]. Prior to the arrival of the Spanish, the Basin of Mexico was inhabited by the Aztec people, who constructed a large city named Tenochtitlan both on and near the lake system. In the early 1600s CE, the Spanish undertook extensive drainage efforts that resulted in the depletion of the lake system, in an attempt to manage and mitigate flooding. The present-day Lake Chalco is a shallow marsh covering an area of < 6 km^2^ in the south of Mexico City [20].

### Stratigraphy and sedimentology

Lake Chalco contains approximately 300 m of lacustrine deposits, which gradually formed through a series of alluvial to fluvial deltaic phases within the Basin of Mexico [19, 21]. The lacustrine deposits of Lake Chalco are estimated to have accumulated over approximately 500,000 years [9, 21]. Sedimentological findings suggest dominant fine-grained lacustrine sediments in the upper 300-meter sediments while lower sediments consist of a volcaniclastic basement [19]. The lacustrine deposits mainly consist of fine-grained sediments including clastic, carbonaceous, diatomaceous, fossil-bearing calcareous mudstone, as well as volcanoclastics [19, 20]. The lacustrine sediments contain 388 individual tephra layers ranging from 0.1 cm to 175 cm in thickness, with an average thickness of 21.8 cm [19]. Among the total 388 tephra layers, about 336 layers (87%) are thicker than 1 cm, 130 tephra layers (33.5%) are thicker than 10 cm, and 108 tephra layers (27.8%) are thicker than 15 cm. In core analysis, tephra layers thicker than 1 cm are readily detectable, while thinner layers are more difficult to separate them from background sedimentation [19].

### Origin of γ-ray signal

**Fig 3:**
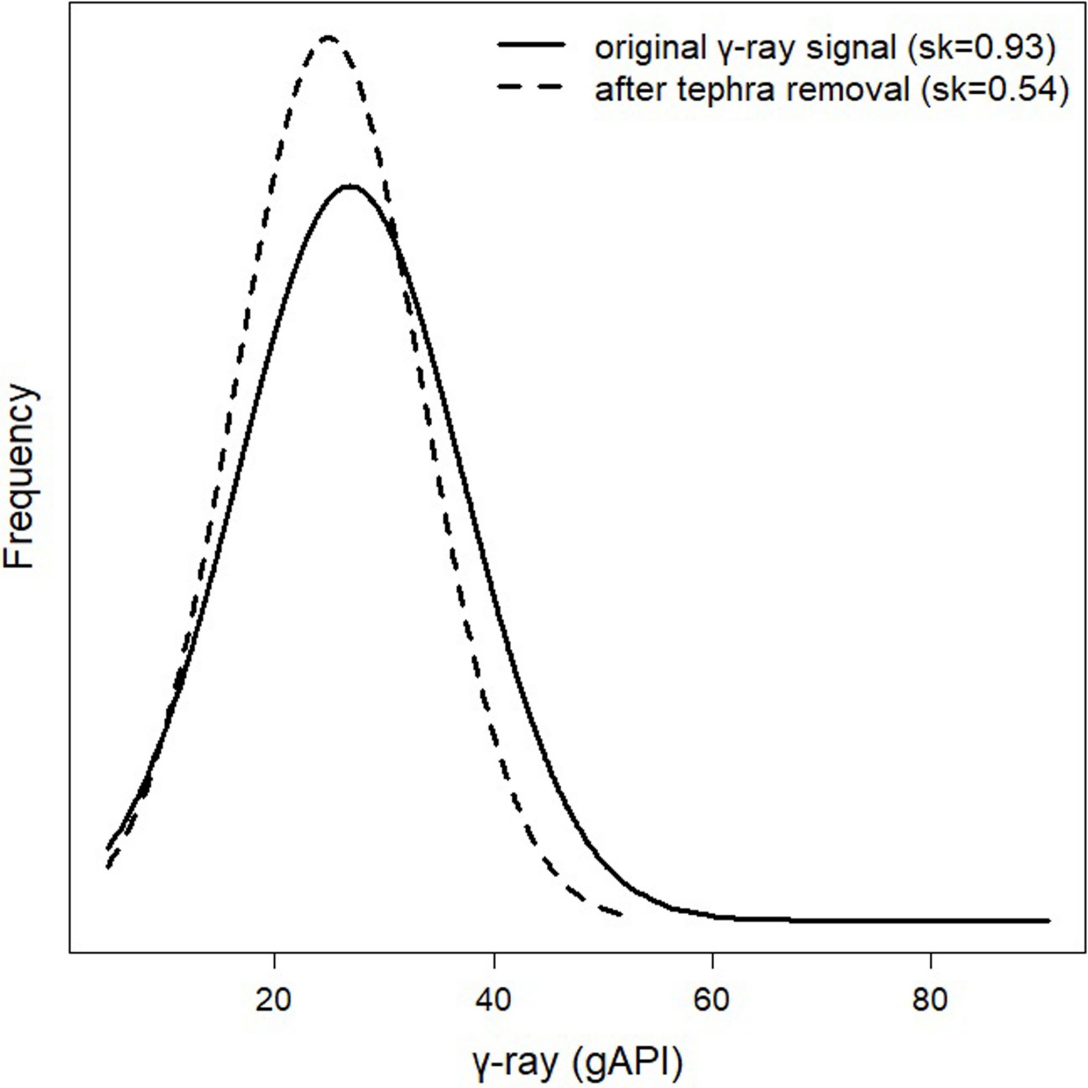
γ-ray signals pre- and post-tephra removal. The distribution of the γ-ray signal measured in borehole MEXI-CHA16-1A of Lake Chalco before (solid line) and after tephra removal (dashed line) from the dataset. The asymmetry of γ-ray distribution is measured as skewness (sk).

Three identified sources of γ-radiation in the sediment of Lake Chalco are (a) detrital input, (b) deposition of volcanic ash as tephra layers and (c) enrichment of authigenic uranium in anoxic sediments [9]. The primary origin of γ-radiation in the lacustrine deposits is detrital sediments carried from weathered volcanic rocks. The distribution of radiogenic elements is influenced by the predominant mechanism of sedimentation, with higher γ-ray values indicating greater detrital input. The unimodal distribution of the γ-ray signal in Lake Chalco indicates the detrital sedimentation resulting from weathering, erosion and transportation process [9] (Fig 3). To a lesser degree, the formation of authigenic uranium due to the dominant redox conditions at the bottom water elevated the concentration of uranium content in the lake sediments. In addition to the detrital sediments, tephra and authigenic uranium are sources of γ-radiation in lake sediments. The right skew observed in the unimodal distribution of the γ-ray signal (Fig 3) can be linked to the presence of both tephra and authigenic uranium in sediments [9]. The γ-ray signals recorded in the lacustrine deposits of Lake Chalco preserved a record of quasi-cyclical components with specific frequencies, highlighting the potential of lacustrine sediments as a climatic archive (S1 Fig) [9]. The tephra layers can interfere with the original climate signals depending on their γ-ray emission intensity. Therefore, removing the effect of tephra layers from the γ-ray signals is expected to improve the original signals associated with climatic responses.

## Materials and methods

The targeted site (MEXI-CHA16-1A; 19°15’25.9"N 98°58’31.8"W) was drilled as part of the ICDP MexiDrill program at the depocenter of Lake Chalco (Expedition ID 5060). The drilling reached a total depth of 423 m in continuous composite depth (CCD), with a core recovery rate of 88% [8]. All downhole logging measurements, including spectral γ-ray (SGR) and Micro-Susceptibility (MS), were carried out by the Leibniz Institute for Applied Geophysics. The SGR tool operates with a vertical resolution of 10 cm logging and at a speed of 3 m/min. The MS probe has a vertical resolution of 2 cm and operates at the same logging of 3 m/min. The SGR measurements were performed in two stages using the SGR70 probe (type 1419), which was deployed through the drill string. The SGR measurements were carried out initially through the upper 180 m and the interval between 423–180 m. The MS data was measured using a Micro-Susceptibility instrument (type 1121) in the open hole. Both SGR and MS probes are manufactured by Antares (Germany). The MS data is adjusted according to the variation in borehole diameter and temperature. To adjust the core depth with the logging depth, we compared the result with the initial 88% recovered core [8]. We established a correlation between the magnetic susceptibility signals of the core and borehole log of the MEXI-CHA16-1A site using the ‘linterp’ function within the ‘astrochron’ R package [22, 23]. Although low values of MS are challenging to compare and may not be comparable, our correlation is based on the high values of magnetic susceptibility, which are present due to tephra layers and appear in both core and logging data (S2–S4 Figs). The ‘lintperp’ function performs a linear interpolation of data between specified tie points. The information in this article on the core, including magnetic susceptibility and the stratigraphic position of tephra layers, was produced by the ICDP MexiDrill campaign, with some of the data already published [8, 19].

We propose a five-step protocol for predicting tephra layers in the upper 180 m of the lacustrine sequence of Lake Chalco. The suggested protocol is based on a linear prediction model on a training dataset including known tephra and non-tephra layers in the lacustrine deposits of Lake Chalco. We calculated similarities between known tephra layers, differences in γ-ray signals between tephra and non-tephra layers, and the composition of energy channels at various depths within a subset of the training dataset. We used multiple linear regression models including stepwise selection as a predictive analysis to explain the relationship between the composition of tephra layers as a dependent variable and the constituent energy channels of the γ-ray signal as independent variables. We calculated the adjusted regression coefficient to scale the R-squared (R^2^) by the number of independent variables in our predicting model and summarized the model reporting F-statistic (F), adjusted R-squared (R^2^), and *P* value. We used a t-test to compare the distribution of tephra and non-tephra groups and summarized the model reporting t-test result (t), degrees of freedom (df) and *P* value. The estimates resulting from linear regression models indicate the coefficients that best represent the relationship between the independent variables (constituent energy channels of the γ-ray signal) and the dependent variable (composition of tephra layers). The R code and two datasets, including the training dataset and the complete γ-ray spectrum data for the studied interval, are provided as S1–S3 Files. The tephra prediction results compared to the distribution of tephra layers that have been reported from the MexiDrill core [8].

To assess the effect of tephra removal on the γ-ray signal, we compared the quality of the cyclical components associated with the γ-ray signal both before and after tephra removal. We applied wavelet analysis to track changes in the regular cycles, aiming to determine whether the cyclical components improved following the removal of tephra from the γ-ray signal [22, 23]. Wavelet transformation provides a hierarchical decomposition of time and frequency (or period) information, allowing us to identify which cyclical components are active or inactive at specific periods across time or depth.

### Five-step protocol for predicting tephra layers

Here we propose an index to identify tephra layers in spectral γ-ray downhole logging data. The tephra index (TI) is comprised of the γ-ray intensity (number of involved channels) and quality (average γ-ray energy) in each signal. We calculated this index in five steps described below.

#### 1) Data validation

**Fig 4:**
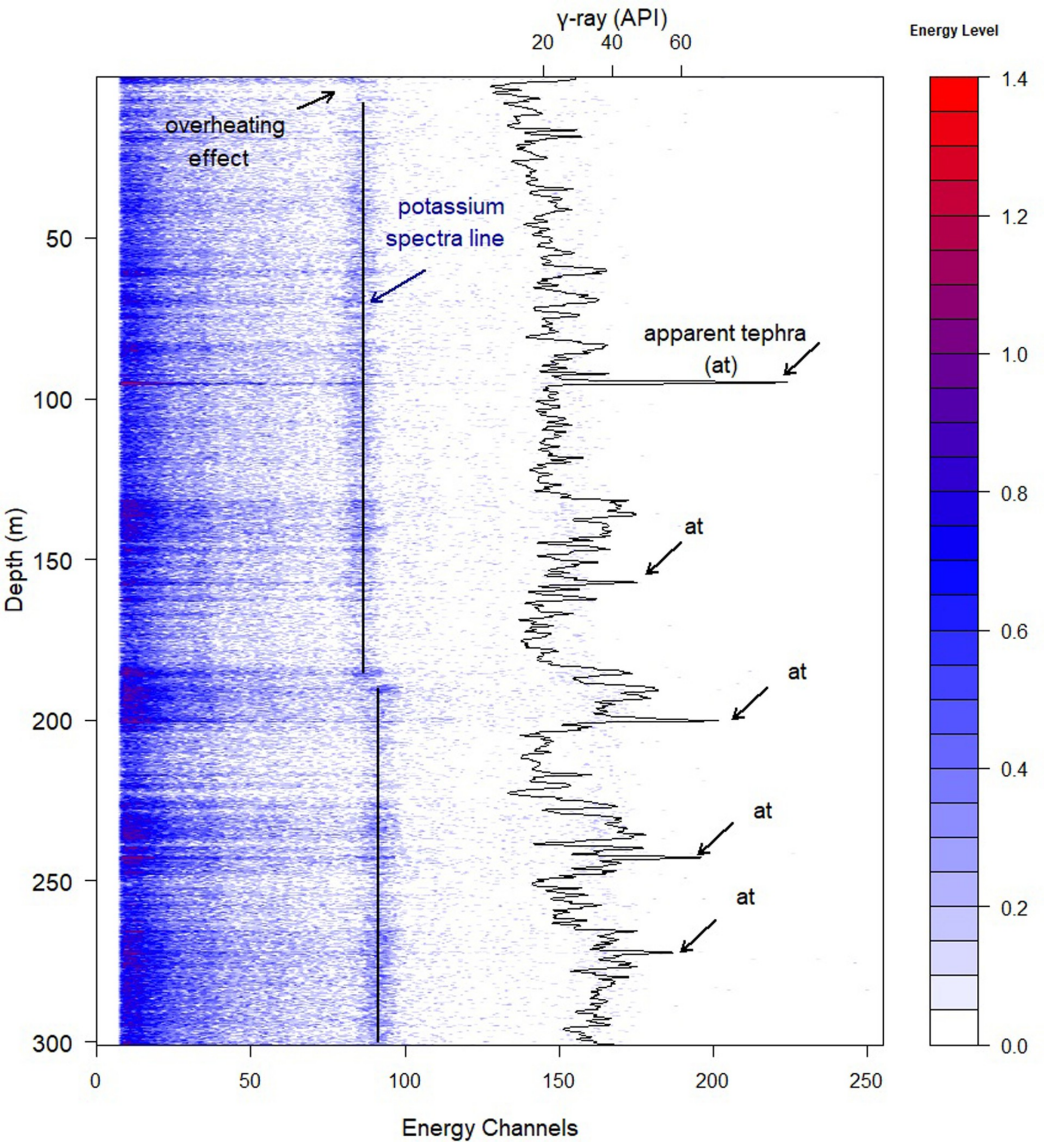
Energy spectrum of the borehole MEXI-CHA16-1A. Depth variation (ordinate) of the energy spectrum of γ-ray photons (abscissa) across the 300 m lacustrine sediment of Lake Chalco. The color bar indicates the level of detected energy. γ-ray signal is additionally illustrated across the depth. Please note that the results of this study focus only on the upper 180 m of Lake Chalco. The results for depths between 180 and 300 m are documented in the S4 File.

The γ-ray spectrum data are framed as a 2-dimensional matrix where the ordinate corresponds to depth and the abscissa corresponds to the channel number representing the γ-ray energy distribution (Fig 4). Values are scaled to the number of recorded photons within a channel and at a specific depth. The initial step is verifying the accuracy of γ-ray spectrum data for consistency in the temporal and spatial distribution of energy photons in the spectrum. In an ideal γ-ray spectrum, the spatial distribution of each isotope takes place in a specific channel energy across the ordinate dimension (depth). For instance, we expect that the potassium-40 isotope (^40^K) displays a characteristic vertical line coinciding with the 1.46 MeV γ-ray or energy channel 85 (S5 Fig). The potassium spectra occur as narrow bands including up to 10 energy channels around channel 85. This is due to the Compton effect where the energies of the original photons are reduced in matter between the source and detector. We suggest creating a contour plot for a γ-ray spectrum dataset to check the temporal (stratigraphic) variation in the potassium spectral line. We can calculate the linear regression of the average γ-ray values of potassium over the depth and use the *P*-value to determine whether γ-ray values are trending over depth (e.g., *P* > 0.05 indicates no trend; *P* < 0.05 indicates the presence of a trend). If the potassium spectral line exhibits a trend, detrending is required before following the next step.

In the case of Lake Chalco, measuring the γ-ray spectrum through the lake at two stages causes a shift in the spectrum at the depth of 180 m (Fig 4). The shift is visually obvious through the potassium spectral line. The potassium spectrum is captured in channel 90 below 180 m but shifts to Channel 85 above 180 m. The observed shift at a depth of 180 m is very likely caused by a change in the temperature of the SGR tool. Therefore, we divided the γ-ray spectrum dataset of Lake Chalco into two matrices, one below and another one above 180 m (Fig 4). Our study represents the results of analyses on the top matrix (above 180 m depth) to avoid redundancy in result presentation. A similar approach is taken for the matrix below 180 m, and the results are presented in S6 and S7 Figs.

The first step is crucial to ensure that the γ-ray spectrum data set accurately represents the matrix or requires editing. Temperature variations can significantly affect the γ-ray measurements by the SGR tool. As temperature varies, the overall gain of the system changes, causing photons to be detected in higher energy channels. The shift is due to the properties of the crystal used in the sensor and the system’s electronics. The energy level of γ-ray photons can be affected either by overheating of the SGR instrument or temperature differences within the borehole. For instance, we excluded the upper 5 m of borehole γ-ray spectrum data from the MEXI-CHA16-1A site due to the effect of our overheating SGR instrument (Fig 4). The potassium spectral line starts increasing at a depth of 5 m, followed by an immediate increase in the γ-ray signal (Fig 4). Temperature variations, regardless of the cause, can result in a general trend in the γ-ray signal.

#### 2) Test which of the spectral γ-ray channels predict tephra layers in a subset of the data with known tephra and non-tephra layers

For this step, we need to select a few depth horizons previously identified as tephra and non-tephra using available information such as geologic reports of other drilling sites of the lake under study. The best strategy is to first focus on apparent tephra layers, which typically exhibit a distinctive signature compared to the background sediments. A contour plot of γ-ray spectrum data can help to identify these tephra layers. For instance, a 15 cm-thick tephra layer was reported at a depth of 96 meters in core MEXI-CHA16-1A, which may appear as a strong, distinctive horizon around the same depth on the γ-ray spectrum contour plot for the borehole at that site (Fig 4). To select non-tephra horizons, it is better to choose layers that either lack obvious tephra signals or correspond to intervals with low tephra occurrence. Geological reports from similar investigation sites or nearby areas are often helpful in this selection process.

Next, the depths from the known tephra and non-tephra layers must be matched with the borehole depths. In our case study, we conduct a comparison between the magnetic susceptibility of the core [8, 19] and borehole to establish a correlation between the depth of the core sediments and the logging depth of the MEXI-CHA16-1A site. Here, we use 20 apparent tephra layers, each over 3 cm in thickness in a core that shows a sharp increase in γ-ray signal through the upper 180 meters of the analyzed borehole MEXI-CHA16-1A (Table 1). It is preferable to use non-tephra horizons adjacent to tephra layers to reduce the effect of radiation on sediments containing tephra (Table 2). The non-tephra should be selected to ensure it is clearly not tephra and represents the greatest variety of lithologies. For example, in the case of Lake Chalco, including the γ-ray spectra of horizons that coincide with plagioclase and feldspar-bearing sands and coarse sediments as non-tephra in the training dataset will enhance the predictive power of the final model.

**Table 1:** Selected tephra layers above 180 m, used in the training dataset.

Depth of representative tephra on log (m)	Depth of identified tephra on core (m) in continuous composite depth (CCD) [19]	Tephra thickness based on core (cm) [19]
4.76	3.64	41.87
7.36	7.16	17.31
8.16	8.68	28.32
17.06	20.08	59
19.16	20.71	35.72
35.36	39.06	41.15
45.36	45.99	22
54.36	58.00	6.22
68.46	69.90	6
70.36	72.20	14.77
92.66	93.22	3
95.26	96.00	15.62
118.96	119.99	21.5
132.16	134.57	9.49
144.56	144.54	19.77
157.56	157.20	119
158.16	158.18	76.87
161.86	161.45	109.57
162.96	162.71	127.50
171.06	171.74	8.12

**Table 2:** Selected non-tephra layers above 180 m, used in the training dataset.

Depth of representative non-tephra on log (m)	Depth of identified non-tephra on core (m) in continuous composite depth (CCD) [19]	Lithology [19]
4.56	8.08	peaty sapropel
16.56	21.95	mottled carbonaceous mud
22.96	29.11	banded carbonaceous mud
52.56	62.27	volcaniclastic
63.66	74.71	banded carbonaceous mud
78.66	91.51	laminated diatomaceous mud
99.96	115.37	banded carbonaceous mud
114.96	132.18	laminated diatomaceous mud
117.86	135.43	calcareous ooze
118.56	136.22	calcareous ooze
122.16	140.25	banded carbonaceous mud
128.26	147.08	peaty sapropel
129.96	148.98	peaty sapropel
131.36	150.55	banded carbonaceous mud
146.16	167.13	banded carbonaceous mud
150.56	172.06	banded carbonaceous mud
159.46	182.03	volcaniclastic
160.96	183.71	banded diatomaceous mud
164.16	187.30	volcaniclastic
176.36	200.970	banded carbonaceous mud

#### 3) Determining which channels will be included in the Tephra Index using stepwise selection

In this step, we select the channels indicative for tephra and non-tephra horizons. First, we specify a model where the predicted variable (y variable) is the identity of a sample (either tephra or non-tephra) and each channel is an individual driver variable (x variable). To avoid zero inflation, it is recommended to only use the channels without large numbers of zeros across the range of depths. Next, we remove channels from the model that are not useful predictors of tephra layers using stepwise selection and the “stepAIC” argument in the R package MASS [24] and using both forward and backward selection in the R software [23].

#### 4) Create a tephra index

We create a tephra index (TI) by multiplying the channels in the stepwise selected model by their estimates and summing the results. Specifically, the tephra index is calculated by summing the predicted values of each channel (Channeln) multiplied by their contributions (Estimates) in the multivariate linear model (Eq 1).


$$Tephra\;Index=\sum_{n=0}^{NC}\;Channel_{n}\times Estimate_{n}$$ (1)

Where NC is the number of used channels.

Where *Estimate* refers to the coefficient determined by the linear regression model for each corresponding energy channel, representing the strength and direction of the relationship between that channel and the tephra index.

The TI quantifies the likelihood that a depth horizon is a tephra layer.

#### 5) Determine the cut-off for the TI between tephra and non-tephra samples

**Fig 5:**
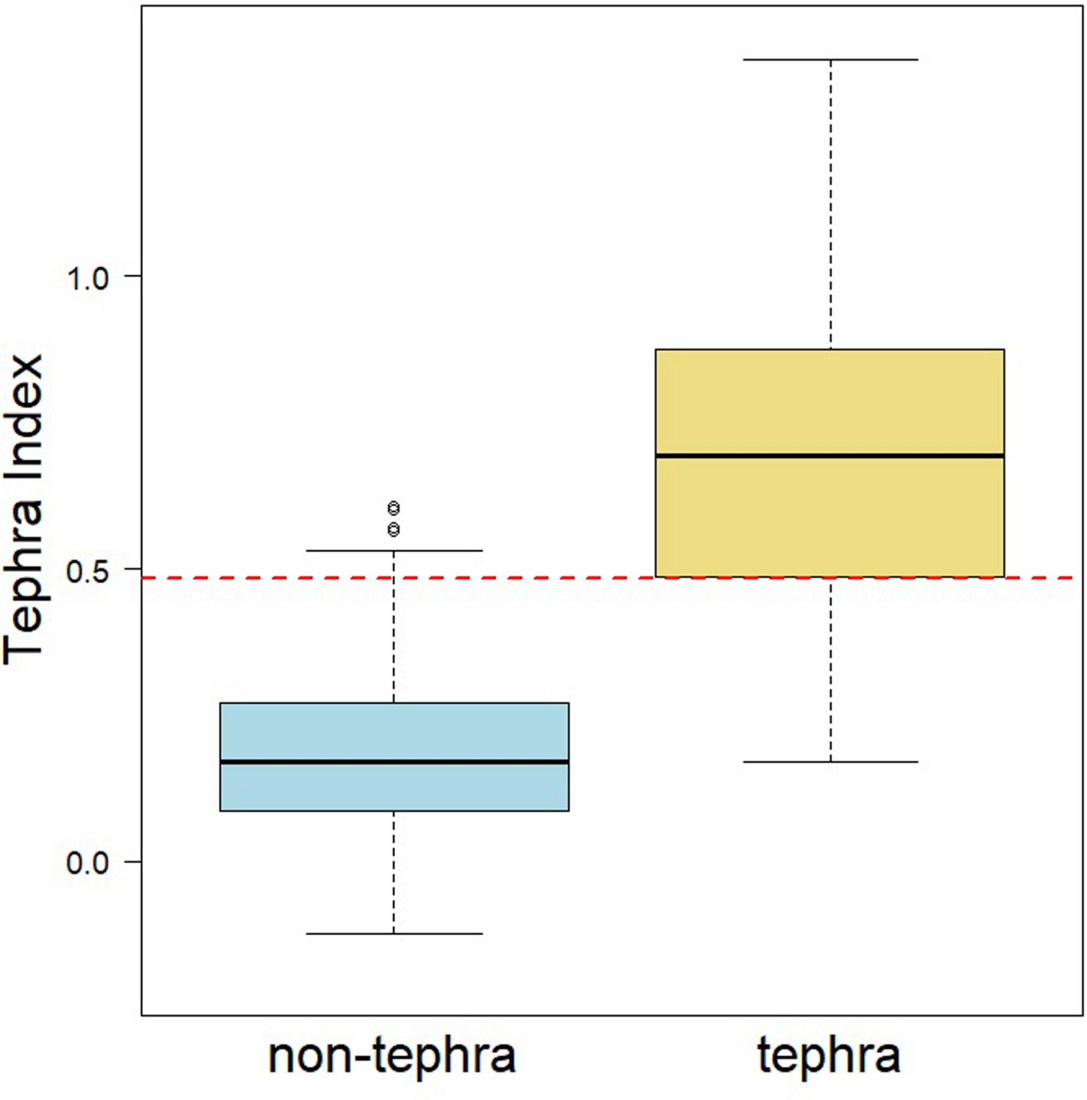
Tephra index distribution for known and non-tephra samples. Boxplot showing the distribution of the tephra index (TI) for the known tephra and non-tephra samples. The cut-off was made at the lower quartile of the tephra distribution (TI = 0.47). The accuracy of this model for the known sample sets is 85%.

To determine a cut-off for the TI, we first visualize the distribution of the TI for the tephra and non-tephra samples using a boxplot. The geological context of the lake system can influence where to apply a cut-off value for identifying tephra versus non-tephra layers. In case the sources of sediments for the lake are not directly supplied from volcanoes or igneous rocks, we can expect two distinct distributions for TI values for tephra and non-tephra samples. Thus, we can identify a clear cut-off value between TI values identified as tephra and TI values identified as non-tephra (Fig 5) which will well-distinguish tephra layers in the remainder of lake sediments. However, for lakes that formed close to volcanic systems, there is more likely to be a range of TI values which overlap between tephra and non-tephra samples. We recommend making the cut-off either at the highest value of the non-tephra distribution or at the lower quartile of the tephra distribution if the distributions overlap. Determining the cut-off at any point across the overlapping range intersected by tephra and non-tephra distribution will add noise so that some non-tephra layers will be detected as tephra layers in the final model. Setting the cut-off must minimize noise in the final model. After conducting all steps, we calculate the TI for all samples/depths, and apply the cut-off to separate the TI-identified tephra and non-tephra horizons (Fig 5).

## Results

### Variation of γ-ray signature in tephra layers

To understand the similarities in the γ-ray signature of known tephra layers, we examined the temporal distribution of three radiogenic elements and the composition of the constituent energy channels of tephra layers across depths. The distribution of Th (sd = 0.7) has higher variation than U (sd = 0.3) or K (sd = 0.2) among the selected tephra layers above 180 m depth. Temporal variation of Th varies slightly with depth (F_(1,43)_ = 20.46, R^2^ = 0.3, *P* < 0.001), while the temporal variation of K (F_(1,43)_ = 3.96, R^2^ = 0.06, *P* = 0.05) and U (F_(1,43)_ = 6.6, R^2^ = 0.11, *P* < 0.001) with depth are less significant. The γ-ray signal of the selected tephra layers indicates ∼59% similarity in the composition of its constituent energy channels across depth (F_(119,45)_ = 6.3, R^2^ = 0.59, *P* < 0.001). Depth variation of tephra layers has no significant effect on the intensity of the γ-ray signal and the composition of its constituent energy channels within the selected tephra layers (F_(1,43)_ = 1.16, R^2^ = 0.13, *P* = 0.64).

### Tephra vs non-tephra

Predicting tephra layers from the background lake sediments is required to get a better understanding of the evident differences between tephra and non-tephra layers in the intensity of the γ-ray signal and the individual arrangement of its energy channels. To assess which of the spectral γ-ray channels predict tephra layers in a subset of the data with known tephra and non-tephra layers, we used linear regression. The results indicate a significant difference in the composition of constituent energy channels between tephra and non-tephra layers (F_(314,83)_ = 4.82, R^2^ = 0.44, *P* < 0.001). We determined 26 channels that have moderately high predictive value for identifying tephra layers using stepwise selection (F_(371,26)_ = 15.67, R^2^ = 0.49, *P* < 0.001) (S1 Table).

### Predicting model

**Fig 6:**
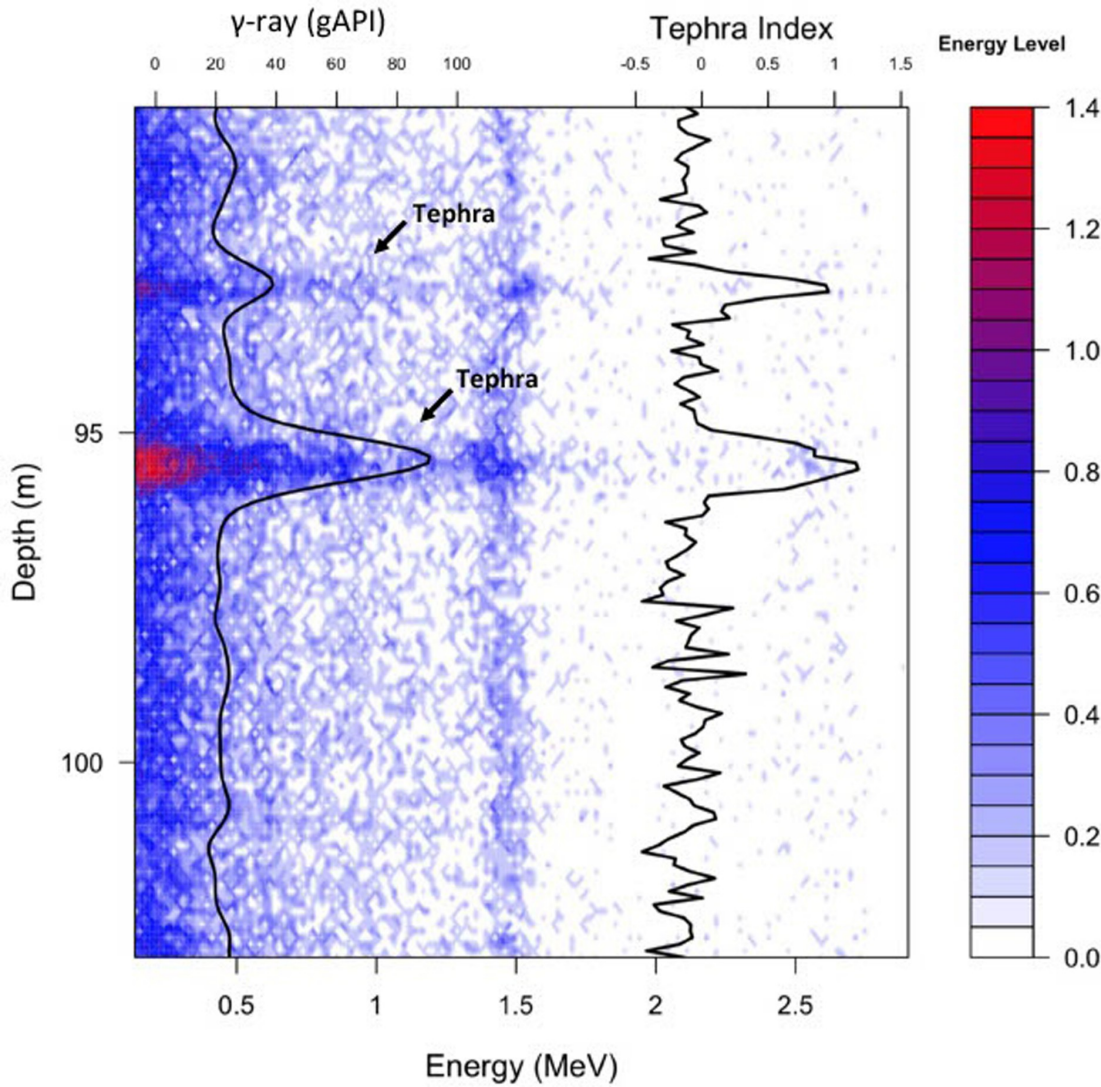
Depth variation of γ-ray photon energy. The contour plot shows the depth variation (ordinate) of the energy spectrum of γ-ray photons (abscissa) between 90 m and 105 m in the borehole MEXI-CHA16-1A. The color bar indicates the level of detected energy. Tephra Index (TI) and γ-ray signal are additionally illustrated across the depth. Two tephra layers with the real thickness of 3 cm and 15 cm predicted by the TI respectively at the depth of 92.6 m and 95.3 m of the spectral γ-ray borehole log.

We developed a tephra predicting model based on the 59% similarities in the composition of tephra layers and the significant differences between the composition of tephra and non-tephra layers through the depth in a subset of the training dataset. We used 26 predictive channels as the independent variables to predict the tephra layers as the dependent variables in our model. In this case, the TI values range between -0.12 and 1.37, where -0.12 indicates the presence of tephra is highly unlikely and 1.37 indicates a high likelihood of a tephra layer (Figs 5 and 6). TI values (mean = 0.70) were larger than non-tephra values (mean = 0.18) in the subset of the training datset (t = -12.86, df = 47, *P* < 0.001).

Tephra and non-tephra distributions overlap in the TI, with 13.3% of tephra layers and 6.7% of non-tephra layers mislabeled. Some overlap is inevitable: cutting the TI to include all tephra layers (TI = 0.17) leads to > 49% of non-tephra layers included as tephra layers; contrary, cutting the TI to remove all non-tephra (TI = 0.54) removes 31% of the tephra layers. We made a cut-off at the lower quartile of the tephra distribution (cut-off for TI = 0.47). With the current cut-off, there is 13% of known tephra layers and 2.8% of known non-tephra layers are misidentified (S8 Fig).

## Discussion

### Methodological considerations regarding fitting

**Fig 7:**
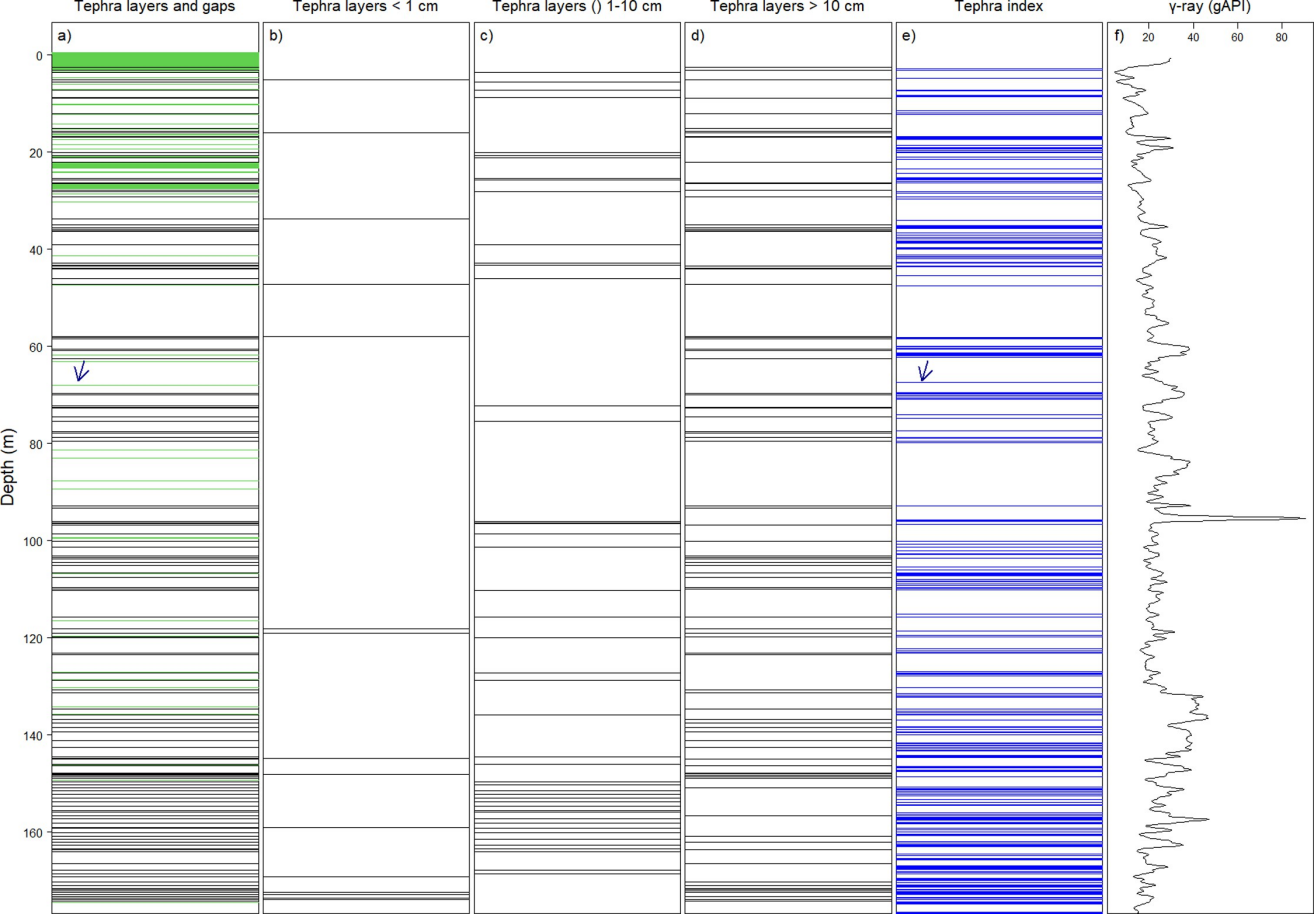
Tephra layer depth: Core samples vs predicted from γ-ray spectrum. Depth distribution of tephra layers from core samples [19] compared to tephra layers predicted from spectral γ-ray log. Panels include: (a) total identified tephra layers (black lines) and gap horizons (green lines) across the upper 180 m of Lake Chalco’s deposits from core sampling (gaps refer to those horizons not recovered during coring and therefore represent regions with no sediment record [19], (b), (c) and (d) distributions of the defined tephra layers filtered based by respective thicknesses of less than 1 cm (< 1 cm), between 1 cm and 10 cm () and greater than 10 cm (> 10 cm), (e) the detected tephra layers based on the calculated tephra index, and (f) the γ-ray signal. An arrow in panel a shows a gap horizon at 68 m depth of the core, and an arrow in panel e at 67.36 m indicates the assumed equivalent TI.

We propose a predictive model to identify if the source of γ-radiation is the tephra layer or lake sediment. In this case, the final model contains 26 channels as predictor variables and has a moderately high predictive value for identifying tephra layers (F_(371,26)_ = 15.67, adjusted R^2^ = 0.49, *P* < 0.001). Using the most predictive channels and their variation in the produced energy of γ-ray photons, our model predicted 85% of tephra layers that could be visually matched with those tephra layers documented in the lake deposits based on core descriptions (Figs 7 and S8). Within the upper 180 m lake deposit, our tephra index detected 142 tephra layers, while the core description of the same borehole reported a total of 160 tephra layers with a minimum thickness of 0.1 cm (Fig 7). Please note that the visual consideration for matching tephra layers identified in the core and the results of the tephra index strongly depends on core-log depth matching. Therefore, matching an exact tephra layer from the core with a representative tephra index based on log data is challenging and associated with uncertainty. Perhaps applying the tephra index to the γ-ray spectrum of the core and comparing it with the identified tephra from the core would result in less uncertainty.

Comparing results between predicted tephra and described tephra layers from the core sampling suggests that the spectral γ-ray tool can be expected to not identify thin tephra layers. The sample rate of 10 cm for the spectral γ-ray tool is expected to identify thick tephra layers (≥ 10 cm in thickness) (Fig 6). The absence of tephra layers in a 10 cm lacustrine interval results in a prediction that sediments are non-tephra. If the tephra layer is less than 10 cm, then the γ-ray value represents a summation of γ-radiation from both the lake sediments and the tephra layers. TI values for tephra layers with ≤ 1 cm in thickness overlap with the values for non-tephra layers (Fig 5). However, the TI values increase in the case of multiple thin tephra layers within a given 10 cm measured band. The presence of several thin tephra layers (≤ 1 cm in thickness) within a 10 cm lacustrine interval results in a prediction that the interval is a tephra (Fig 7). When lake sediments are dominant, they conceal thin embedded tephra layers within the 10 cm intervals. The latter case coincides with an overlap in the γ-ray distributions of tephra and lacustrine sediments.

### Geological considerations

The composition of clastic material among the lake sediments affects the degree of overlap in the γ-ray distributions of tephra and lacustrine sediments. Coarse-grained volcaniclastics (≥ 1 mm in diameter) very likely produce similar γ-ray signals matching the composition of tephra layers. A combination of grain size data and core description regarding the core-log integration can be used to recognize the beds containing coarse-grained volcaniclastics sediment from tephra layers. Clastic sediments of Lake Chalco are mainly composed of clay and silt sized fragments (≤ 63 μm) transported from surrounding volcanic rocks in the hinterland. The dominance of fine-grained clastic sediments suggests that the original mineralogical composition of the precursor sediment may not be preserved due to the strong mechanical and chemical weathering during transport from source to sink.

We expected that the degree of overlap in the γ-ray distributions of tephra and lacustrine sediments is minimal where the lake sediments are not sourced directly by adjacent volcanic rocks. In this case, the lake sediments contain volcanic tephras from different origins which are likely different in their γ-ray signature. To improve the predictor variables of TI to predict tephra layers from different origins or eruption phases, we recommend including tephra layers from different depths in the borehole into training datasets. We can increase the efficiency of the TI to differentiate the thin layers of tephra from the background lacustrine sediments by decreasing the logging speed and improving the performance of Bismuth Germanate Oxide (BGO). Large crystal of BGO detects more γ-ray photons and increase the quality of the signal [25]. However, if the goal of a study is to use a γ-ray signal for interpretation of paleoclimate and paleoenvironment, then the presence of thin tephra layers not recorded by the γ-ray signal will not result in additional noise that must first be removed.

### Relevance of removing tephra

**Fig 8:**
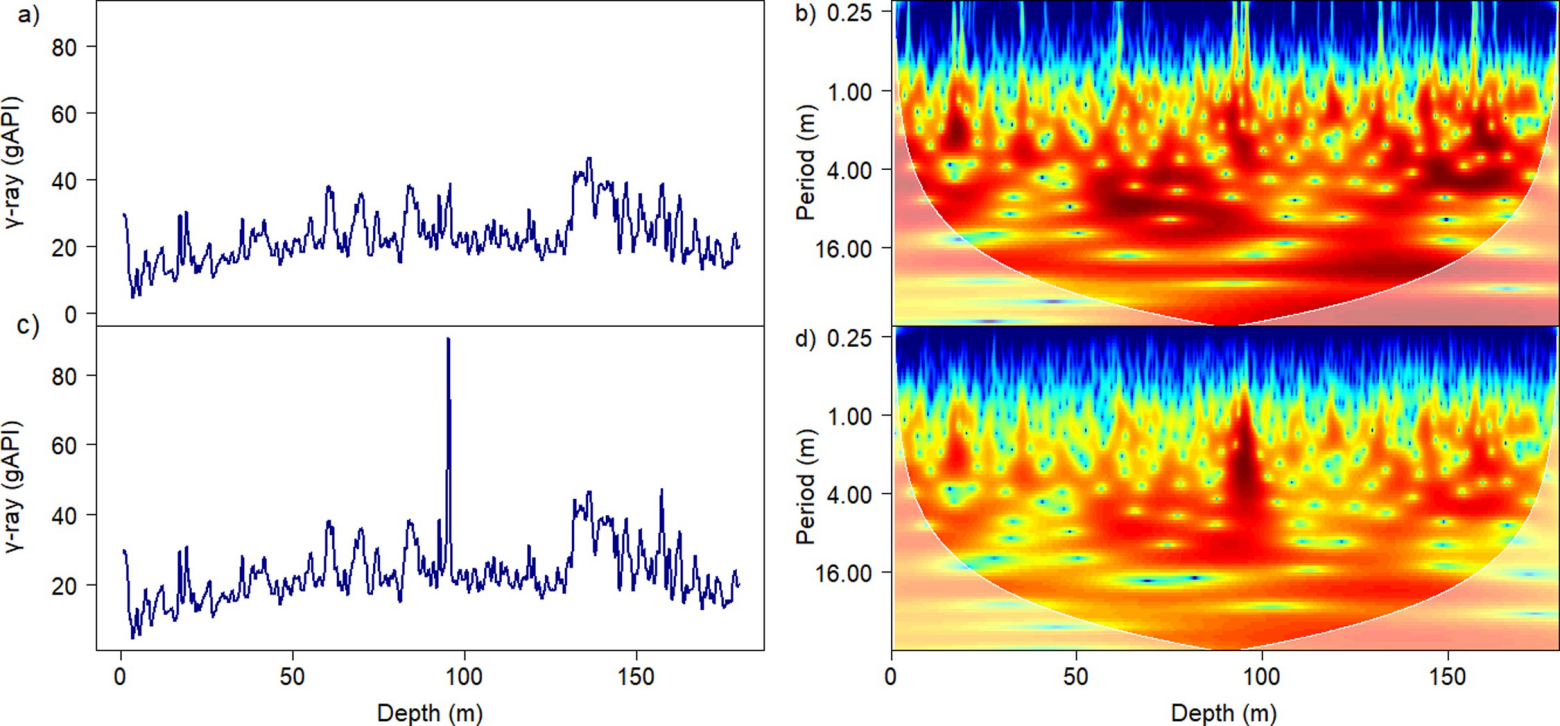
The cyclical components associated with the γ-ray signal before and after tephra removal. Wavelet analysis (panel “b” after and panel “d” before removal of the tephra layers) of the detrended γ-ray signal (panel “a” after and panel “c” before removal of tephra layers) was recorded across the lacustrine deposits of Lake Chalco. The color code in the wavelet analysis indicates high cyclicity (red) and low cyclicity (blue) for depth (abscissa) and different periodicity (ordinate). White shading highlights parts not fully covered by data.

By excluding the γ-ray values associated with the predicted tephra layers from the γ-ray time series, it became evident that the cyclical patterns within the γ-ray signal display more distinct periodicity (Fig 8). The capability of acquisition of γ-ray in a cased hole allows the TI to have potential for detecting tephra layers that may not be present in cores (in the case of the MEXI-CHA16-1A site, the core recovery is 88%). For instance, the TI diagnosed several horizons within the 180 meters of the studied borehole where the equivalent sediment sequence was not completely recovered during the drilling operation. The layers within the stratigraphic interval that coincide with the horizons missed during coring are predicted to be tephra if their TI values are higher than 0.61 (as TI > 0.61 is the highest value for non-tephra samples in the subset of the training dataset). For instance, our TI identified a tephra layer (TI > 0.61) at the 67.36 m interval of the γ-ray borehole log. In contrast, the equivalent interval in the MexiDrill core at 68 m coincides with a gap horizon with a thickness of 17 cm reported within the same interval [8]. We hypothesize that the a tephra layer predicted by our TI are missing in the equivalent horizon of the MexiDrill core due to the presence of a gap at 68 m interval (Fig 7).

### Value for stratigraphy

We can use the TI to distinguish tephra layers in a borehole, facilitating targeted sampling through wall samples for further analysis. The distribution of tephra layers in sediments is linked to other geological proxies, resulting in improved lithostratigraphic correlation in an interior basin [26]. The stratigraphic location of tephra layers discloses the various stages of volcanic activity in a region [27]. The distribution of tephra layers in sediments plays a critical role in constructing more precise and accurate age-depth models, which are essential for comprehending past environmental changes and the evolution of sedimentary basins [28]. Detecting and assigning tephra layers to eruptions with known age can contribute to age-depth models. In this regard, our TI index provides the stratigraphic position of tephra layers in a borehole. The integration of temporal correlation of tephra layers with other stratigraphic tools (such as chemostratigraphy and biostratigraphy) creates comprehensive event stratigraphy [4].

In contrast, when using an astrochronological method to evaluate climate-related trends, it is crucial to exclude tephra layers from the proxy data [28]. With the aid of our TI, we can remove these layers systematically and dependably, effectively eliminating their impact on the analysis. By removing tephra layers from our dataset, the skewness of the γ-ray distribution was significantly reduced (Figs 3 and 8). To derive the climate signal from (lacustrine) sediments, it is simpler to interpret a distribution that is symmetric or nearly symmetric than one that is skewed. More specifically, decreasing the skewness minimizes the negative influence of outliers on regression-based models, resulting in a continuous high-frequency cycle associated with γ-ray signals [9].

### Comparison of TI and conventional tephra detection methods

Conventional tephra detection methods are broadly developed on several intrinsic physical and chemical characteristics, often crossing multiple scientific disciplines [12, 13]. Traditionally, physical properties such as grain size, color, and thickness have been frequently used to visually identify tephra layers [11, 15]. However, to detect thinner or less obvious tephra layers, more advanced techniques have been developed that focus on mineralogical composition and geochemical signatures (e.g., XRF, ICP-MS) [14]. While conventional methods can provide highly accurate and detailed information at micrometer scales, they rely on physical access to the sample [29]. This is a limitation when compared to the TI method, which does not require physical sampling, as discussed in this contribution.

In traditional methods for detecting tephra layers across stratigraphic sequences using borehole geophysical data, magnetic susceptibility and γ-ray logs are typically combined [28]. However, this traditional approach heavily relies on core descriptions, which are obtained by visually comparing the core with the log data during core-log integration. The quality of the core, along with the thickness and frequency of tephra layers, significantly influences the effectiveness of these traditional techniques.

The current TI technique introduces a novel approach for predicting tephra layer locations in borehole logs, independent of core samples. TI relies on the strength and composition of the γ-ray signal to identify embedded tephra layers. However, there are some limitations to this technique that need to be considered. The accuracy of TI predictions strongly depends on the quality of γ-ray spectrometry data (as discussed in step 1) and the robustness of the training datasets (steps 2:4). Compositional variation among tephra layers, due to differences in eruption events over time, can affect the γ-ray signatures in these layers. Including tephra layers from various depths can mitigate this issue.

Another important factor in predicting tephra layers using TI is the compositional contrast between tephra and the surrounding sediments. The clearer the difference between the two, the more accurate and reliable the TI method becomes. Additionally, the resolution of γ-ray measurements during borehole logging plays a key role in tephra prediction accuracy. Although a vertical logging resolution of 10 cm is considered high-resolution for SGR datasets, it still cannot match the resolution of conventional methods based on core description and -samples, which are capable of detecting cryptotephra thinner than 1 mm in thickness.

## Conclusions

This study assesses the hypothesis that tephra layers exhibit a distinct γ-ray spectrum that is representative of their composition. The distribution of photons emitted from a tephra layer across the energy spectrum channels can be considered an intrinsic characteristic of that tephra. This distinct spectrum enables their differentiation from adjacent sedimentary deposits. The γ-ray spectrum comparison results for 20 tephra layers within the upper 180 meters of the lacustrine deposits of Lake Chalco reveal a 59% similarity in the distribution pattern of energy photons across the spectrum. The 41% variation in γ-ray spectrum patterns among the 20 tephra layers likely indicates that different eruption phases have distinct γ-ray signatures. The difference between the γ-ray spectra of the tephra layers and lacustrine sediments (non-tephra) is statistically significant. The significant difference between the γ-ray signatures of tephra and lake sediments at Lake Chalco is used to automatically predict the presence of tephra within lacustrine deposits using γ-ray spectroscopy. Removing the γ-ray values of predicted tephra layers from the γ-ray time series has revealed that the cyclical components of the γ-ray signal exhibit clearer cyclicity.

We propose a five-step protocol to determine the presence of tephra layers derived from a natural γ-ray signal from lacustrine deposits. The first step, which is only necessary if the γ-ray spectrum shows a general trend, is to detrend the entire γ-ray spectrum. In the second step, we select several horizons that have been identified as either tephra or non-tephra layers during core sampling of lake sediments. After selecting the known horizons (tephra vs. non-tephra) in step two, step three consists of a series of procedures to determine the energy channels that are most indicative for the presence of tephra layers and to incorporate these energy channels into a multivariate regression model. In the fourth step, a tephra index (TI) is created by multiplying the relevant channels by their multivariate regression estimates and summing them. Finally, the TI expresses the probability that a horizon consists of a tephra layer. Visualization of the TI for selected tephra and non-tephra horizons from a training dataset aids in the application of a threshold for distinguishing tephra from non-tephra. In the case of Lake Chalco, the TI allows prediction of 85% of the tephra layers in the upper 180 m interval consisting of lacustrine sediments. Therefore, the model we have developed has the potential to be used for identifying tephra layers in various geological settings.

## Supporting information

S1 File R code. This R script is used for the TI and includes steps 2 to 5 of the protocol.
(R)

S2 File Training dataset. The attached CSV file, titled ’Tephra_nonTephra.csv,’ contains the training dataset used in this study.
(CSV)

S3 File Complete γ-ray spectrum data. The Excel file titled ’LCH_SPC.xlsx’ contains the complete γ-ray spectrum data for the studied interval.
(XLSX)

S4 File List of 20 selected tephra layers used in the training dataset. This dataset includes the section ID of each individual tephra layer selected for the training dataset above 180 meters.
(XLSX)

S1 Fig γ-ray signal in lake Chalco deposits pre- and post-tephra removal. γ-ray signal across the lacustrine deposits of Lake Chalco before (bottom) and after (top) removing tephra layers. Our TI detected 363 tephra layers, while 388 total tephra layers were reported from the core description of the same borehole. Five apparent tephra layers (>10 cm in thickness) are indicated by black arrows.
(DOCX)

S2 Fig Core-log depth correlation using magnetic susceptibility. Correlation between core depth (upper plot) and logging depth (lower plot) using magnetic susceptibility signals of the core and borehole log.
(DOCX)

S3 Fig Correlation between core depth and log depth. This figure illustrates the correlation between tie points of core depth and logging depth, using magnetic susceptibility signals from the core and borehole log. Please note that core depth is measured in continuous composite depth (CCD). The corresponding tie points are listed in the following table.
(DOCX)

S4 Fig Core depth aligned with log depth. The top plot shows the magnetic susceptibility signal recorded from the core, with the core depth aligned to the log depth. The bottom plot presents the magnetic susceptibility signal recorded from the borehole log along the log depth. Both plots illustrate the correlation between core and log depth, enabling a direct comparison of the magnetic susceptibility signals.
(DOCX)

S5 Fig Correlation between the range of γ-ray spectrum and energy channels in a γ-ray spectrum. The ordinate represents the depth variation of the energy spectrum of γ-ray photons. The abscissa lower axis shows the energy of γ-ray photons, while the abscissa upper axis represents the energy channels of the γ-ray spectrum across the 300-meter lacustrine sediment of Lake Chalco. The color bar indicates the detected energy levels. Additionally, the γ-ray signal is illustrated across the depth.
(DOCX)

S6 Fig Tephra Index for depth 180 and 300 m of Lake Chalco sediments. Boxplot showing the distribution of the TI (Index) for the known tephra and non-tephra samples. The cutoff made at the highest value of the non-tephra distribution (Index = 0.40). The accuracy of this model for the known sample sets is 96.5%.
(DOCX)

S7 Fig Depth distribution of tephra layers recorded from core sampling versus the detected tephra layers. Panels include: (a) total identified tephra layers (black lines) and gap horizons (red lines) across the between 180 and 300 m of the Lake Chalco’s deposits from core sampling (gaps refer to those horizons not recovered during coring and therefore represent regions with no sediment record), (b, c and d) distributions of the defined tephra layers filtered based by respective thicknesses of 1 cm, between 1 cm and 10 cm and thicker than 10 cm, (e) the detected tephra layers based on the calculated Tephra Index, and (f) the γ-ray signal.
(DOCX)

S8 Fig Evaluation of the training data set. Using a cut-off value of 0.47 resulted in 13% of known tephra layers being misidentified.
(DOCX)

S1 Table Summary model for stepwise regression. The list of 26 channels is the best predictor of tephra layers compared to lake sediments.
(DOCX)
