# Supplementary material for: Predicting the presence of tephra layers in lacustrine deposits using spectral gamma ray data: An example from Lake Chalco, Mexico City
Source: PLoS One. 2024 Dec 30;19(12):e0315331. doi: 10.1371/journal.pone.0315331 (PMC11684696; doi:10.1371/journal.pone.0315331)
Supplement: S1 Fig — γ-ray signal across the lacustrine deposits of Lake Chalco before (bottom) and after (top) removing tephra layers. Our TI detected 363 tephra layers, while 388 total tephra layers were reported from the core description of the same borehole. Five apparent tephra layers (>10 cm in thickness) are indicated by black arrows. (DOCX) [file pone.0315331.s005.docx]

**Supporting figure 1:**


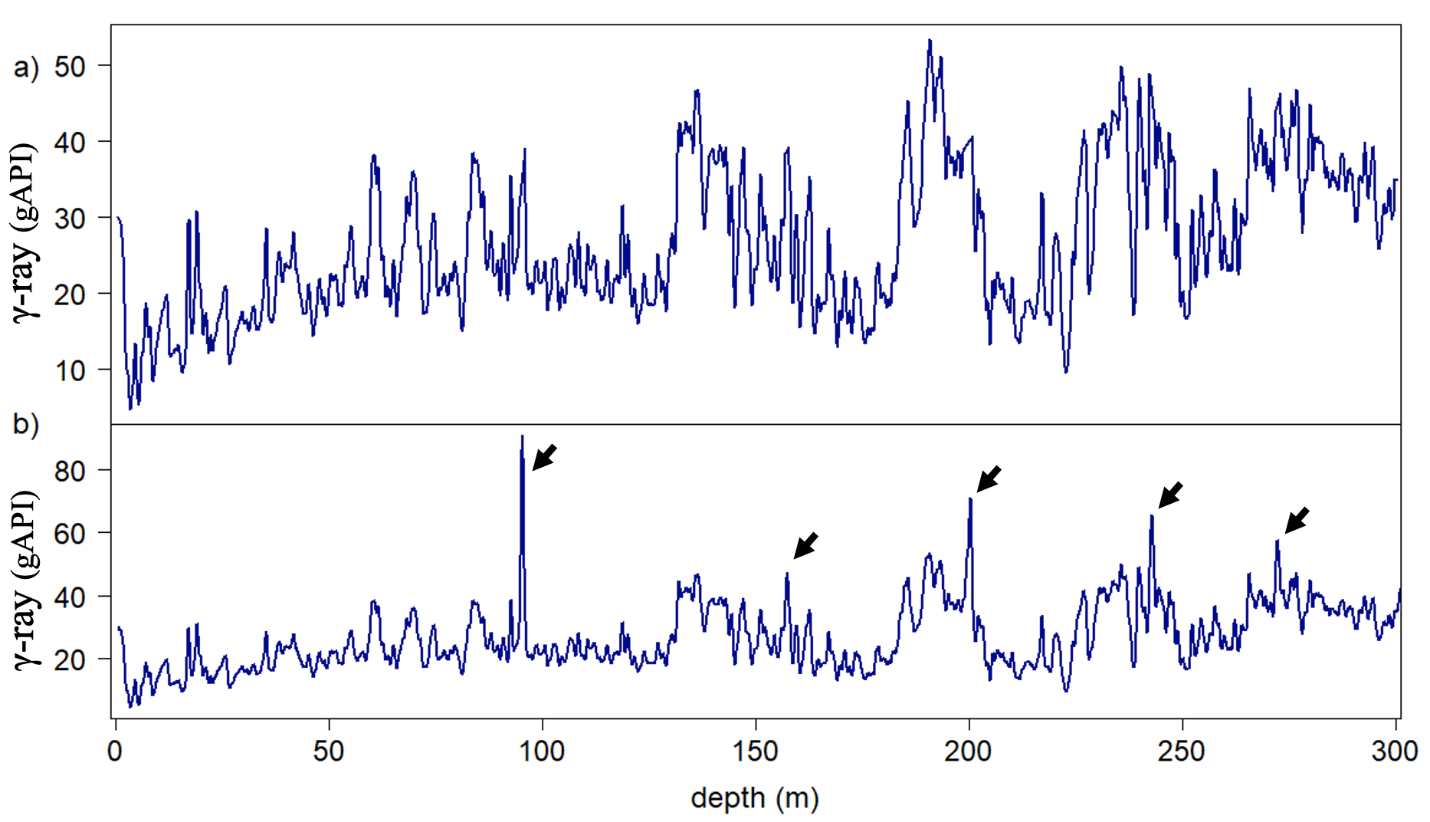


**S1 Fig. γ-ray signal in lake Chalco deposits pre- and post-tephra removal.** γ-ray signal across the lacustrine deposits of Lake Chalco before (bottom) and after (top) removing tephra layers. Our TI detected 363 tephra layers, while 388 total tephra layers were reported from the core description of the same borehole. Five apparent tephra layers (>10 cm in thickness) are indicated by black arrows.
