# Supplementary material for: Predicting the presence of tephra layers in lacustrine deposits using spectral gamma ray data: An example from Lake Chalco, Mexico City
Source: PLoS One. 2024 Dec 30;19(12):e0315331. doi: 10.1371/journal.pone.0315331 (PMC11684696; doi:10.1371/journal.pone.0315331)
Supplement: S2 Fig — Correlation between core depth (upper plot) and logging depth (lower plot) using magnetic susceptibility signals of the core and borehole log. (DOCX) [file pone.0315331.s006.docx]

**Supporting figure 2:**

**
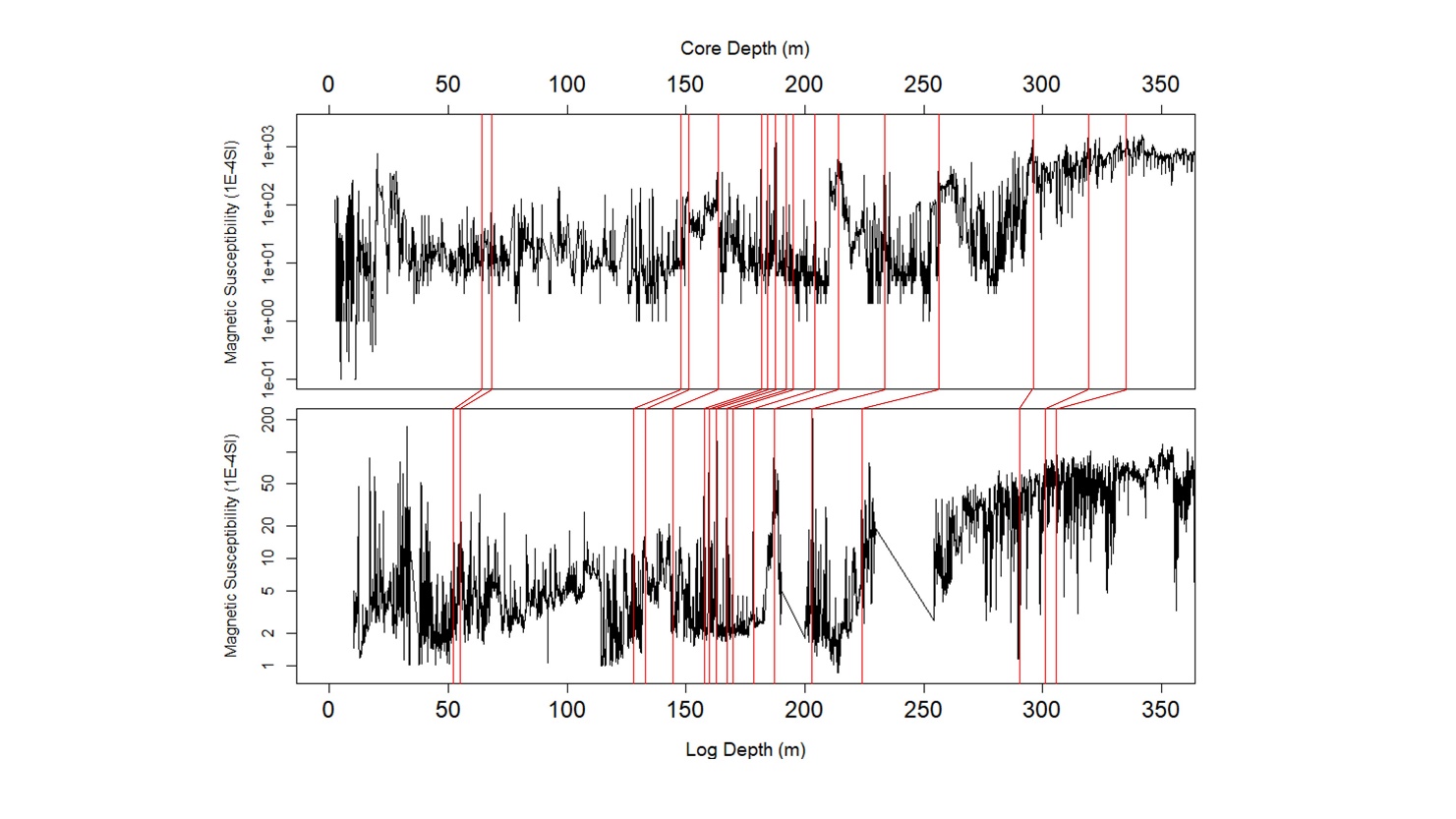
**

**S2 Fig. Core-log depth correlation using magnetic susceptibility.** Correlation between core depth (upper plot) and logging depth (lower plot) using magnetic susceptibility signals of the core and borehole log.
