# Supplementary material for: Predicting the presence of tephra layers in lacustrine deposits using spectral gamma ray data: An example from Lake Chalco, Mexico City
Source: PLoS One. 2024 Dec 30;19(12):e0315331. doi: 10.1371/journal.pone.0315331 (PMC11684696; doi:10.1371/journal.pone.0315331)
Supplement: S3 Fig — This figure illustrates the correlation between tie points of core depth and logging depth, using magnetic susceptibility signals from the core and borehole log. Please note that core depth is measured in continuous composite depth (CCD). The corresponding tie points are listed in the following table. (DOCX) [file pone.0315331.s007.docx]

**Supporting figure 3:**


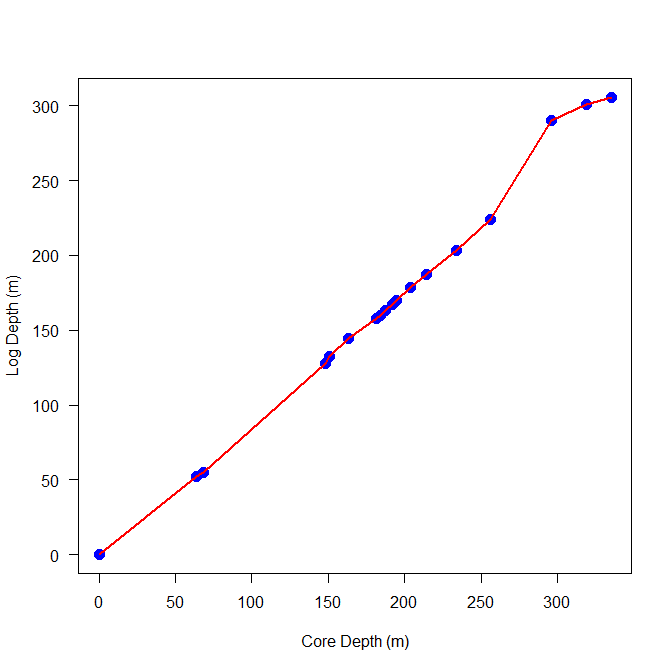


**S3 Fig. Correlation between core depth and log depth.** This figure illustrates the correlation between tie points of core depth and logging depth, using magnetic susceptibility signals from the core and borehole log. Please note that core depth is measured in continuous composite depth (CCD). The corresponding tie points are listed in the following table:

| Depth_core (m) | Depth_log (m) |
| --- | --- |
| 0 | 0 |
| 63.943 | 52.26 |
| 68.188 | 54.86 |
| 147.877 | 127.86 |
| 150.918 | 132.76 |
| 163.357 | 144.56 |
| 181.59 | 157.56 |
| 184.178 | 159.96 |
| 187.518 | 162.86 |
| 192.112 | 167.26 |
| 194.9 | 169.66 |
| 204.077 | 178.26 |
| 214.088 | 187.26 |
| 233.639 | 203.06 |
| 256.123 | 224.06 |
| 296.011 | 290.16 |
| 319.132 | 300.96 |
| 335.153 | 305.76 |
