# Supplementary material for: Predicting the presence of tephra layers in lacustrine deposits using spectral gamma ray data: An example from Lake Chalco, Mexico City
Source: PLoS One. 2024 Dec 30;19(12):e0315331. doi: 10.1371/journal.pone.0315331 (PMC11684696; doi:10.1371/journal.pone.0315331)
Supplement: S4 Fig — The top plot shows the magnetic susceptibility signal recorded from the core, with the core depth aligned to the log depth. The bottom plot presents the magnetic susceptibility signal recorded from the borehole log along the log depth. Both plots illustrate the correlation between core and log depth, enabling a direct comparison of the magnetic susceptibility signals. (DOCX) [file pone.0315331.s008.docx]

**Supporting figure 4:**

**
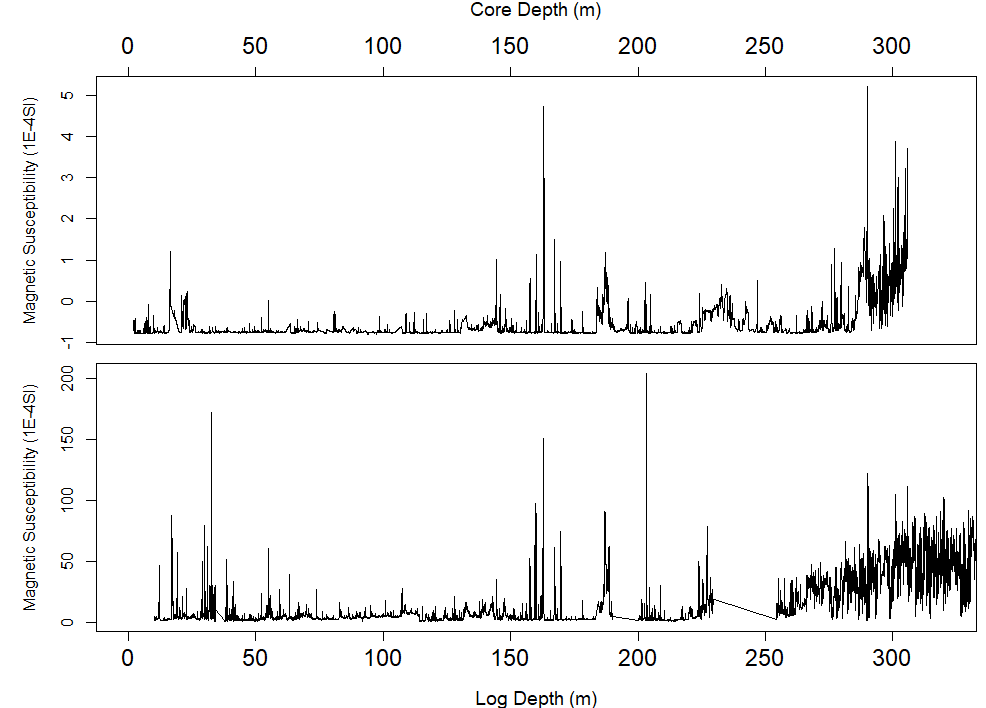
**

**S4 Fig. Core depth aligned with log depth.** The top plot shows the magnetic susceptibility signal recorded from the core, with the core depth aligned to the log depth. The bottom plot presents the magnetic susceptibility signal recorded from the borehole log along the log depth. Both plots illustrate the correlation between core and log depth, enabling a direct comparison of the magnetic susceptibility signals.
