# Supplementary material for: Predicting the presence of tephra layers in lacustrine deposits using spectral gamma ray data: An example from Lake Chalco, Mexico City
Source: PLoS One. 2024 Dec 30;19(12):e0315331. doi: 10.1371/journal.pone.0315331 (PMC11684696; doi:10.1371/journal.pone.0315331)
Supplement: S5 Fig — The ordinate represents the depth variation of the energy spectrum of γ-ray photons. The abscissa lower axis shows the energy of γ-ray photons, while the abscissa upper axis represents the energy channels of the γ-ray spectrum across the 300-meter lacustrine sediment of Lake Chalco. The color bar indicates the detected energy levels. Additionally, the γ-ray signal is illustrated across the depth. (DOCX) [file pone.0315331.s009.docx]

**Supporting figure 5:**


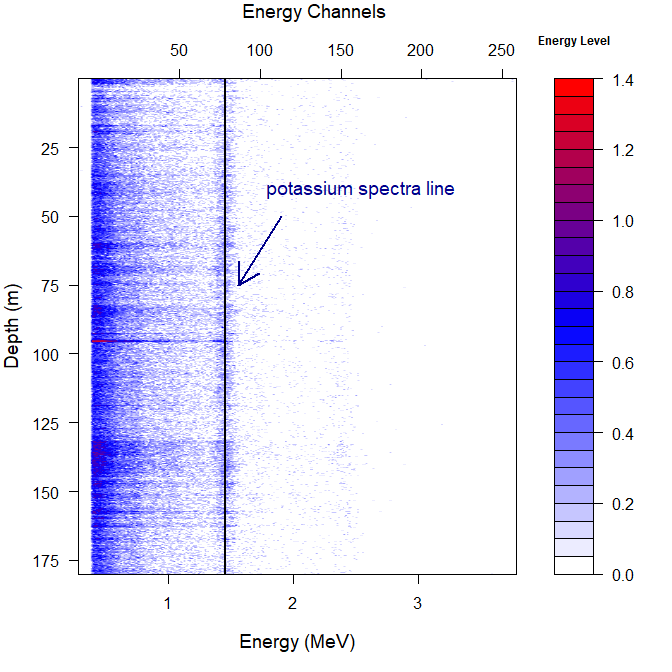


**S5 Fig. Correlation between the range of γ-ray spectrum and energy channels in a γ-ray spectrum.** The ordinate represents the depth variation of the energy spectrum of γ-ray photons. The abscissa lower axis shows the energy of γ-ray photons, while the abscissa upper axis represents the energy channels of the γ-ray spectrum across the 300-meter lacustrine sediment of Lake Chalco. The color bar indicates the detected energy levels. Additionally, the γ-ray signal is illustrated across the depth.
