# Supplementary material for: Predicting the presence of tephra layers in lacustrine deposits using spectral gamma ray data: An example from Lake Chalco, Mexico City
Source: PLoS One. 2024 Dec 30;19(12):e0315331. doi: 10.1371/journal.pone.0315331 (PMC11684696; doi:10.1371/journal.pone.0315331)
Supplement: S6 Fig — Boxplot showing the distribution of the TI (Index) for the known tephra and non-tephra samples. The cutoff made at the highest value of the non-tephra distribution (Index = 0.40). The accuracy of this model for the known sample sets is 96.5%. (DOCX) [file pone.0315331.s010.docx]

**Supporting figure 6:**

**S6 Fig. Tephra Index for depth 180 and 300 m of Lake Chalco sediments.** Boxplot showing the distribution of the TI (Index) for the known tephra and non-tephra samples. The cut-off made at the highest value of the non-tephra distribution (Index = 0.40). The accuracy of this model for the known sample sets is 96.5 %.
