# Supplementary material for: Predicting the presence of tephra layers in lacustrine deposits using spectral gamma ray data: An example from Lake Chalco, Mexico City
Source: PLoS One. 2024 Dec 30;19(12):e0315331. doi: 10.1371/journal.pone.0315331 (PMC11684696; doi:10.1371/journal.pone.0315331)
Supplement: S7 Fig — Panels include: (a) total identified tephra layers (black lines) and gap horizons (red lines) across the between 180 and 300 m of the Lake Chalco’s deposits from core sampling (gaps refer to those horizons not recovered during coring and therefore represent regions with no sediment record), (b, c and d) distributions of the defined tephra layers filtered based by respective thicknesses of 1 cm, between 1 cm and 10 cm and thicker than 10 cm, (e) the detected tephra layers based on the calculated Tephra Index, and (f) the γ-ray signal. (DOCX) [file pone.0315331.s011.docx]

**Supporting figure 7:**


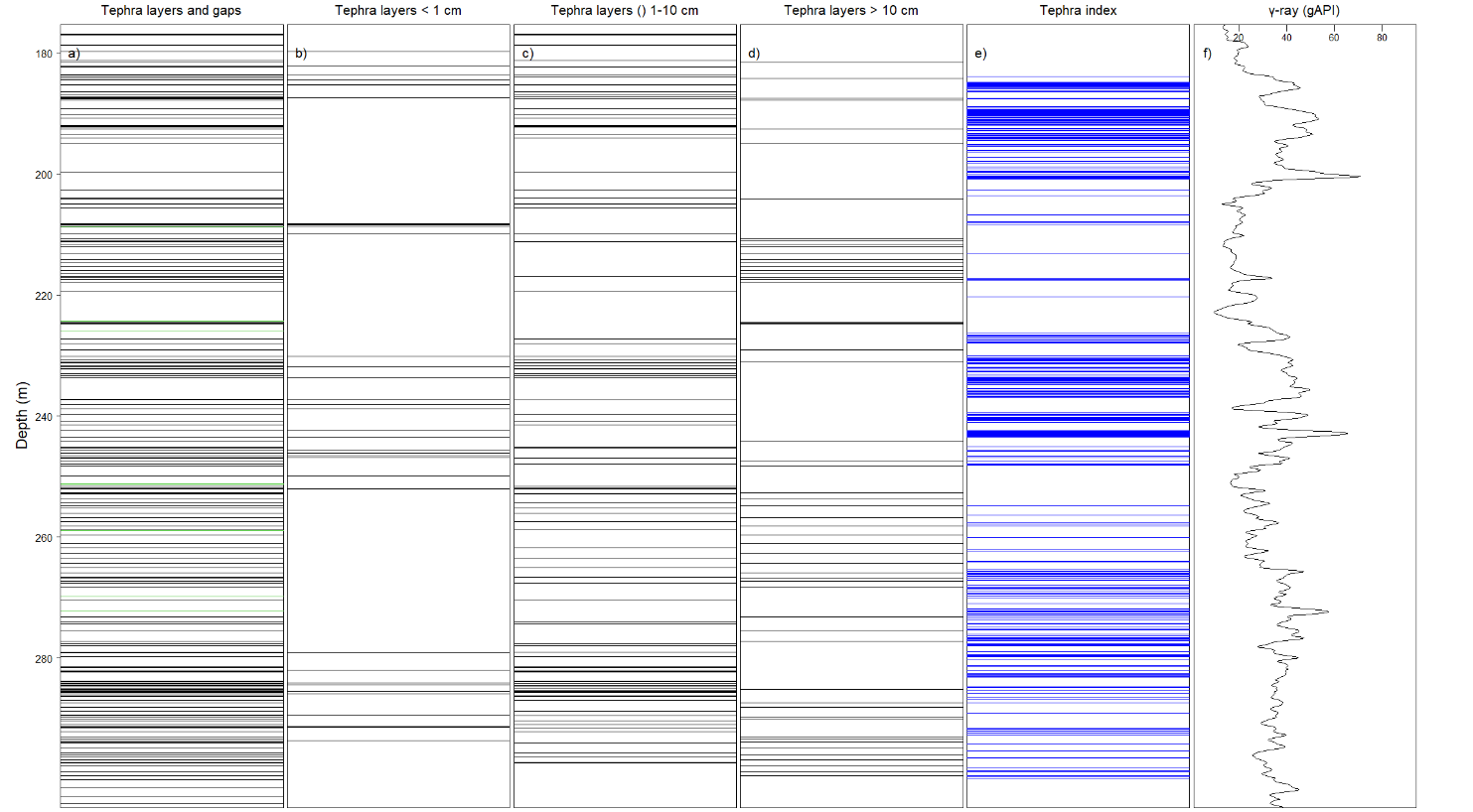


**S7 Fig. Depth distribution of tephra layers recorded from core sampling versus the detected tephra layers.** Panels include: (a) total identified tephra layers (black lines) and gap horizons (red lines) across the between 180 and 300 m of the Lake Chalco’s deposits from core sampling (gaps refer to those horizons not recovered during coring and therefore represent regions with no sediment record), (b, c and d) distributions of the defined tephra layers filtered based by respective thicknesses of 1 cm, between 1 cm and 10 cm and thicker than 10 cm, (e) the detected tephra layers based on the calculated Tephra Index, and (f) the γ-ray signal. A depth offset in panels a-d occurs at ca. 220 m in response the depth shifting of the core data.
