# Supplementary material for: Predicting the presence of tephra layers in lacustrine deposits using spectral gamma ray data: An example from Lake Chalco, Mexico City
Source: PLoS One. 2024 Dec 30;19(12):e0315331. doi: 10.1371/journal.pone.0315331 (PMC11684696; doi:10.1371/journal.pone.0315331)
Supplement: S8 Fig — Using a cut-off value of 0.47 resulted in 13% of known tephra layers being misidentified. (DOCX) [file pone.0315331.s012.docx]

**Supporting figure 8:**

**
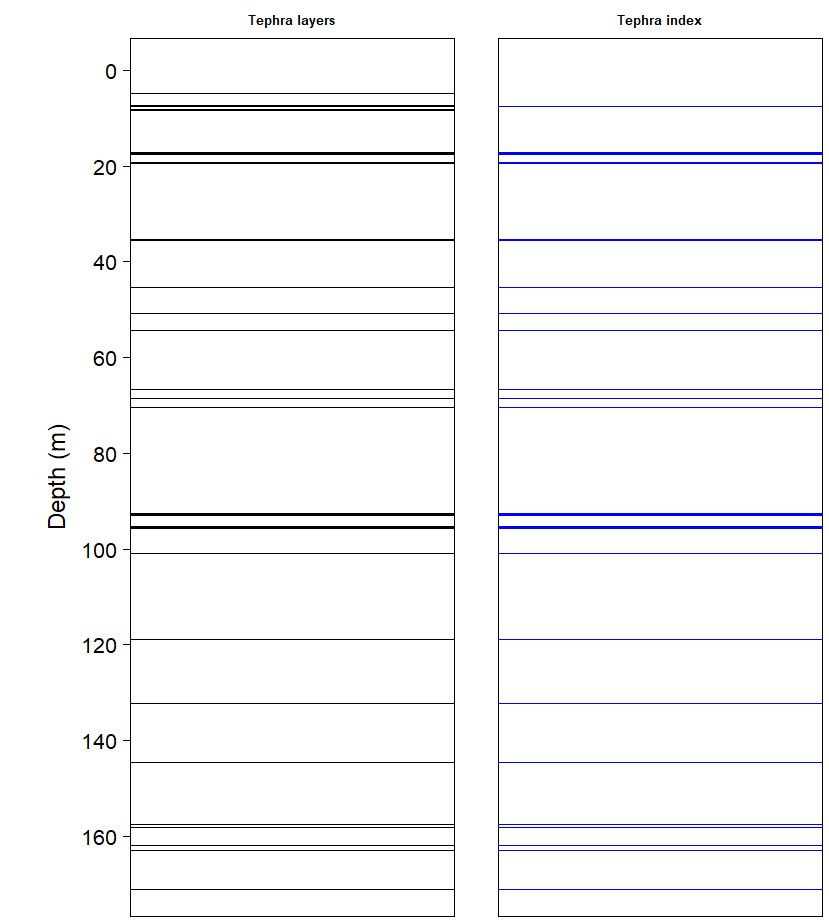
**

S8 Fig. Evaluation of the training data set. With the cut off =0.47 resulted that 13 % of known tephra layers are misidentified.
