# Supplementary material for: Predicting the presence of tephra layers in lacustrine deposits using spectral gamma ray data: An example from Lake Chalco, Mexico City
Source: PLoS One. 2024 Dec 30;19(12):e0315331. doi: 10.1371/journal.pone.0315331 (PMC11684696; doi:10.1371/journal.pone.0315331)
Supplement: S1 Table — The list of 26 channels is the best predictor of tephra layers compared to lake sediments. (DOCX) [file pone.0315331.s013.docx]

| Channel NO. | Estimate | Standard Error | t value | *P* (>\|t\|) |
| --- | --- | --- | --- | --- |
| CHANNEL 8 | 0.088818 | 0.01047 | 8.483 | 5.30E-16 |
| CHANNEL 9 | 0.024992 | 0.008498 | 2.941 | 0.003477 |
| CHANNEL 13 | -0.03173 | 0.009151 | -3.467 | 0.000587 |
| CHANNEL 18 | 0.029582 | 0.012145 | 2.436 | 0.015335 |
| CHANNEL 19 | -0.02174 | 0.011259 | -1.931 | 0.054221 |
| CHANNEL 24 | -0.02632 | 0.015329 | -1.717 | 0.086825 |
| CHANNEL 28 | -0.02262 | 0.015378 | -1.471 | 0.142097 |
| CHANNEL 36 | -0.02631 | 0.016114 | -1.633 | 0.103379 |
| CHANNEL 39 | 0.05855 | 0.021964 | 2.666 | 0.008017 |
| CHANNEL 41 | 0.052834 | 0.02326 | 2.271 | 0.023693 |
| CHANNEL 46 | 0.047581 | 0.024796 | 1.919 | 0.055765 |
| CHANNEL 57 | -0.03785 | 0.026824 | -1.411 | 0.159062 |
| CHANNEL 58 | 0.052896 | 0.027261 | 1.94 | 0.053093 |
| CHANNEL 60 | -0.04261 | 0.029492 | -1.445 | 0.149365 |
| CHANNEL 62 | 0.056108 | 0.028055 | 2 | 0.046233 |
| CHANNEL 69 | 0.048183 | 0.030565 | 1.576 | 0.115782 |
| CHANNEL 73 | 0.065826 | 0.036896 | 1.784 | 0.075225 |
| CHANNEL 76 | -0.08502 | 0.034479 | -2.466 | 0.014125 |
| CHANNEL 77 | 0.086649 | 0.036147 | 2.397 | 0.017019 |
| CHANNEL 79 | 0.042164 | 0.027862 | 1.513 | 0.131061 |
| CHANNEL 82 | -0.03808 | 0.023662 | -1.609 | 0.108374 |
| CHANNEL 84 | -0.03037 | 0.020382 | -1.49 | 0.137102 |
| CHANNEL 87 | 0.031698 | 0.022302 | 1.421 | 0.156067 |
| CHANNEL 88 | 0.039782 | 0.023274 | 1.709 | 0.088237 |
| CHANNEL 89 | 0.034756 | 0.023224 | 1.497 | 0.135361 |
| CHANNEL 42 | -0.03257 | 0.023237 | -1.402 | 0.161844 |

S1 Table. Summary model for stepwise regression. The list of 26 channels a sbest predictor of Tephra layers in compare to lake sediments.
